# Dr. Woodville, on Vaccine Inoculation

**Published:** 1801-01

**Authors:** William Woodville

**Affiliations:** Ely Place


### *To the Editors of the Medical and Phyſical Journal,*

Gentlemen,

As the inoculation of the Cow-pox is known to be conducted upon a very extenſive ſcale at the Inoculation Hoſpital, and as the advantages which this new practice poſſeſſes are only to be learned from experience, I have hitherto judged it proper, from time to time, to furniſh the public, not only with the general reſults, but with moſt of the principal facts that have occurred to me in the propagation of this diſeaſe.—It appears, from from my laſt publication on this ſubject, written about ſix months ago, that the number of perſons who had then received the vaccine infection at the Hoſpital exceeded 2500; ſince that time upwards of 1500 have been inoculated for the Cow-pox at the ſame place, and of theſe I have a report to preſent ſimilar to that ſtated by me in July laſt, viz. "With none of the patients did the infection occaſion a ſevere diſorder, or excite one alarming ſymptom."—The number of puſtular caſes under the Vaccine Inoculation, in the Hoſpital, has been even leſs than three or four out of an hundred, the proportion in which ſuch caſes were ſtated to occur at the period above mentioned. Reſpecting thoſe to whom I have communicated the infection out of the Hoſpital, or among my private patients, I have not yet met with one inſtance in which variolous-like puſtules took place. Indeed, I am convinced an eruption of that appearance will be found to be a very rare occurrence, unleſs, previouſly to the Vaccine Inoculation, or during its local progreſs, the patient has been expoſed to the action of variolous matter. Though ſuch an expoſure may not have been known, nor even ſuſpected to have taken place, yet this will not be deemed an objection of much weight againſt the opinion here advanced, when it is conſidered that the ſame obſervation will apply to four-fifths of all who caſually receive the Small-pox.—If a perſon who has been expoſed to the contagion of the Small-pox, for four or five days, be then inoculated for this diſeaſe, the inoculation anticipates, or prevents, the effects of the contagion, and the inoculated Small-pox is produced. But, if the Vaccine Inoculation be employed in a caſe thus circumſtanced, the Small-pox is not prevented, although the tumour produced by the inoculation advance to maturation. Hence we are to expect that the caſual Small-pox will more often ſupervene to the vaccine than to the variolous inoculation.

It was not before the commencement of the preſent year that I aſcertained the Cow-pox had not the power of ſuperceding the Small-pox; for though, from the firſt trials I made of the new inoculation, it appeared that theſe diſeaſes, as produced in the ſame ſubject from inoculation, did not interrupt the progreſs of each other, yet as the caſual does not act in the ſame manner as the inoculated Small-pox, and may be anticipated by the latter, I thought it ſtill probable that the Cow-pock infection might have a ſimilar effect. Numerous facts have, however, proved this opinion to be unfounded, and that the variolous effluvia, even after the Vaccine Inoculation has made a conſiderable progreſs, have, in ſeveral inſtances, occaſioned an eruption reſembling that of the Small-pox. This latter latter effect of the Small-pox I did not conceive to be poffible, till after I had made repeated trials of the new inoculation out of the Hofpital; nor is the fact to be eafily explained, when it is confidered that the vaccine inoculation imparts its effects to the conftitution in a fhorter time than the latent period of variolous infection, which is commonly from the eleventh till the fourteenth day.

In thofe cafes of Vaccine Inoculation in which the variolous infection has an early effect, I have obferved that the tumour at the inoculated part proceeds flowly, and never exhibits any efflorefcence; the puftules alfo are more numerous, when they appear early in the difeafe, than when they do not appear till after the twelfth day of the inoculation.

From the preceding obfervations we may infer, that in this metropolis, and its vicinity, where the Small-pox conftantly more or lefs prevails, the Vaccine Inoculation muft fometimes be attended with a puftular eruption, of which it is not the caufe. But inoculators, not adverting to this, have generally afcribed the eruption to a variolated ftate of the Cow-pock matter, with which the patient was inoculated; and the Inoculation Hofpital has been commonly reprefented as the place in which this adulterated matter was generated and obtained. To refute this opinion I adduced feveral facts, proving that varioliform puftules had frequently accompanied the Cow-pock inoculation, though no doubt could be entertained of the genuinenefs or purity of the vaccine matter employed for the inoculation; and alfo a number of experiments, fufficient to fhew that the Cow-pox does not *hybridife* with the Small-pox, but that both difeafes continue diftinct in the fame patient; of which the following fingular inftance may be confidered as an additional proof.

About two months ago, a girl, eleven years of age, was admitted into the Inoculation Hofpital, where fhe was inoculated with vaccine matter; five days afterwards fhe was feized with the fymptoms of Small-pox, and an eruption of puftules (about 200) took place. On the 10th day of the inoculation, one of the variolous puftules appeared diftinctly within the margin of the vaccine tumour. I charged a lancet with matter taken from the centre of the tumour, and with it inoculated a child, in whom it produced a regular cafe of Cow-pox. Mr. Wachfel, the apothecary to the Hofpital, who inoculated three children with matter taken from the puftule in the vaccine tumour, found that it communicated the Small-pox to all of them.

*Ely Place, Dec.* 1800. WILLIAM WOODVILLE.

